# U.S. public perceptions of the sensitivity of brain data

**DOI:** 10.1093/jlb/lsad032

**Published:** 2024-01-18

**Authors:** Shenyang Huang, Umika Paul, Shikhar Gupta, Karan Desai, Melinda Guo, Jennifer Jung, Beatrice Capestany, William D Krenzer, Dylan Stonecipher, Nita Farahany

**Affiliations:** Center for Cognitive Neuroscience, Duke University, Durham, North Carolina, USA; Duke Initiative for Science & Society, Durham, North Carolina, USA; Duke Initiative for Science & Society, Durham, North Carolina, USA; University of Massachusetts Chan Medical School, Worcester, Massachusetts, USA; Duke Initiative for Science & Society, Durham, North Carolina, USA; Duke Initiative for Science & Society, Durham, North Carolina, USA; University of Michigan Medical School, Ann Arbor, Michigan, USA; Duke Initiative for Science & Society, Durham, North Carolina, USA; University of Massachusetts Chan Medical School, Worcester, Massachusetts, USA; Duke Initiative for Science & Society, Durham, North Carolina, USA; Duke Initiative for Science & Society, Durham, North Carolina, USA; Duke Initiative for Science & Society, Durham, North Carolina, USA; Duke University, Durham, North Carolina, USA; Duke Initiative for Science & Society, Durham, North Carolina, USA; Duke University, Durham, North Carolina, USA

**Keywords:** neuroscience, brain data, neural data, neurotechnology, BCI, privacy, mental privacy

## Abstract

As we approach an era of potentially widespread consumer neurotechnology, scholars and organizations worldwide have started to raise concerns about the data privacy issues these devices will present. Notably absent in these discussions is empirical evidence about how the public perceives that same information. This article presents the results of a nationwide survey on public perceptions of brain data, to inform discussions of law and policy regarding brain data governance. The survey reveals that the public may perceive certain brain data as less sensitive than other ‘private’ information, like social security numbers, but more sensitive than some ‘public’ information, like media preferences. The findings also reveal that not all inferences about mental experiences may be perceived as equally sensitive, and perhaps not all data should be treated alike in ethical and policy discussions. An enhanced understanding of public perceptions of brain data could advance the development of ethical and legal norms concerning consumer neurotechnology.

## I. INTRODUCTION

In the age of rapidly advancing technologies, we are witnessing an era of ‘big data’, characterized by the expansive use of Internet and sophisticated artificial intelligence systems to amass large data sets.^1^ The healthcare sector, in particular, stands to benefit immensely from this trend, given the wide-ranging nature and potential applications of data in both biomedical research and commercial settings.^2^ This paper zeroes in on the intriguing realm of ‘brain data’ and aims to shed light on public perceptions of the sensitivity of this data, especially compared to other types of personal data.

Over the past decade, direct-to-consumer neurotechnologies have started to become increasing available and more popular.^3^ Portable non-invasive electroencephalogram (EEG) devices and electromyography (EMG) devices can now be directly purchased by consumers for a wide range of applications.^4^ These devices enable the monitoring of attention,^5^ the measurement of stress,^6^ and seamless interaction with the virtual world^7^ through the recording and analysis of brainwaves and electrical impulses from muscles. The rising trend of Brain Computer Interfaces (BCI) is noteworthy, appealing to a vast demographic from aiding those with disabilities to enhancing the gaming experience for enthusiasts. They cater to individuals with disabilities as well as everyday users in the gaming and entertainment sectors.^8^ Neurotechnology also has the ability to collect personal data beyond biological brain function, such as individuals’ emotional,^9^ mental, and affective states, and even human thoughts.^10^ More sensitive, private information, like whether an individual has a substance abuse disorder or specific numeric combinations a person is thinking of (ie an untold PIN),^11^ can also be extracted using brainwave data.^12^ In the not-too-distant future, these devices may become one of the primary ways in which we interact with the rest of our technology, including how we drive our cars,^13^ play video games,^14^ and interact with our environment.^15^

With these advances come potential privacy risks associated with the collection and use of neural data. The potential for misuse is not just theoretical, but already being realized, in settings from the workplace to government use of neurotechnologies for criminal interrogation.^16^

Neupane et al. demonstrate how *Hemorrhage*—a term they used when an attack on brainwave privacy is conducted by machine-learning software—analyzed publicly available EEG datasets and predicted the presence of alcohol usage disorder with 96% precision.^17^ While this technique was carried out under controlled settings, real-life consequences of *Hemorrhage* can include blackmail, targeted medical or insurance scams, and serious career implications.^18^ AlterEgo, a headphone that detects silent speech using neuromuscular signals, has an interface with 92% accuracy for digit recognition, which could lead to password leaks.^19^ Finally, as Kellmeyer and others have remarked, there is the potential for de-anonymization of brain data.^20^ MRI data can be used to reconstruct an individual’s face which could ultimately lead to re-identification.^21^ While ‘big brain data’ collected and sold by EEG device manufacturers is anonymized, the possibility of subject re-identification becomes more likely when the personalized features of brain data are cross-referenced with other types of data.^22^ When such data is de-anonymized, it could expose individuals to the risk of identity theft or criminal acts^23^ and be utilized for commercial and marketing purposes.^24^ In light of recent neural data leaks from corporations such as DeepMind and Facebook, there is a need for closer monitoring on the private sector’s use of brain data.^25^

### I.A. Academic Perceptions of Consumer Neurotech Data

In recent decades, there has been a significant increase in the literature addressing the challenges of collecting various forms of neural information using consumer neurotech devices. On one side of the debate, numerous studies sound the alarm about the processing of sensitive neural data and the risks for human rights violations. Other scholars cite the sensitivity of the data itself, the location of the data within corporations, and the risk of inadvertent exposure of this information as motivating concerns.^26^ Professor Farahany has described mental privacy as the ‘final frontier of privacy’,^27^ while Marcello Ienca describes ‘brain information’ as ‘the most intimate and private of all information’,^28^ and Donald Kennedy, the former editor-in-chief of *Science*, describes brain data as constituting his ‘most intimate identity’.^29^ On its own, brain activity data may be able to reveal whether one is attentive or distracted,^30^ as well as drowsy or alert.^31^ In research settings, neurotech devices have shown the potential to measure emotional responses,^32^ identify likes and dislikes, and even assist in the diagnosis of depression^33^ and ADHD.^34^ On a larger scale, the raw data collected by EEGs may even be capable of augmenting already existing algorithms that use personal data like browsing history to target advertisements, calculate insurance premiums, or match potential partners.^35^

A prevailing concern among scholars is the potential for neurotech users to unwittingly overshare. They might not fully grasp the myriad ways in which their data can be harnessed. Farahany reveals numerous instances in which consumers have already started to do so.^36^ Ienca et al. argue that many users may underestimate the informational richness and versatility of brain data, leaving them vulnerable to unintentionally surrendering their data privacy rights.^37^ Consumers may unknowingly authorize a company which uses their data to gain deep insights into their personal profiles.^38^ This enhances the risk of de-anonymization and public exposure.^39^

Brain data is already being commodified by consumer neurotechnology companies, creating a risk of exposure without users’ consent. Farahany describes how one major neurotech company, Entertech, has already entered into partnerships with other companies to share the brain data they have aggregated through use of their consumer EEG devices.^40^ Kellmeyer describes the act of ‘neurohacking’, which he defines as the ‘illicit access to a neurotechnological device or a software program that processes neural data’.^41^ A hacker could hypothetically take over a BCI system connected to a robotic prosthetic and cause grave physical harm to the user and others.^42^ Ienca et al. point to the Cambridge Analytica scandal that engulfed Facebook in 2018 as an example of the dangers associated with loosely regulated data collection.^43^ According to them, the scandal, in which the personal data of 87 million Facebook users was shared without their consent, showed that online providers such as Facebook often lack the willingness and capability to restrict data collection, which enables third parties to access data without authorization.^44^ While there is a push to treat neural data with the same legal protections afforded to other sensitive data such as health data, achieving this has proven challenging due to the inherent uncertainty in its processing as discussed by Hallinan et al.^45^ Coaxes and Wexler assert that the attention on maintaining privacy distracts us from more pressing issues such as the misleading advertising on EEG devices.^46^ Another dimension of this issue was examined by Gerber et al. in a survey in which participants were asked to rank different risk scenarios in terms of severity and probability of data leaks.^47^ The findings showed that abstract, hypothetical scenarios involving their health were considered less alarming when compared to specific scenarios.^48^ This further suggests that the public is not fully informed about data privacy risks and that perceptions vary based on imminent threat, emphasizing the need for a rise in public awareness.^49^ People may willingly share their data with organizations such as insurance providers, employers, and law enforcement agencies that may have a deliberate interest in monitoring people.^50^

On the other side of the debate, there is also the argument that with the development of data regulations and safeguards, the benefits of large-scale brain data sharing and consumption outweigh the concerns. Jwa et al. state that it may be unrealistic to completely eliminate the risk of neural data breaches and more efforts should be placed on prevention measures than safeguarding privacy, which ultimately limits the rewards of neuroscience research.^51^ The potential benefits of large-scale brain data extend to both medical and non-medical sectors. Such data has the capacity to drive advancements in therapeutic medical solutions and the quality of lifesaving neurotechnological devices. Amidst these promising possibilities, concerns exist regarding the effectiveness of consumer neural devices. Wexler and Reiner have argued that there is a lack of scientific consensus on the efficacy of such devices^52^. Rather than enforcing more restrictions, they encourage more comprehensive consumer evaluations and testing to foster the growth and increased reliability of neurotechnology.^53^

### I.B. Industry Perceptions of Consumer Neurotech Data

The perception of brain data collection risks is not uniform even among industry leaders in neurotechnology. A study by Minielly et al. sought to understand these leaders’ views on scholarly privacy concerns.^54^ Minielly et al. interviewed senior executives from prominent neurowearable firms and distilled their insights into four primary themes: data collection and management, ethical principles, the unique nature of brain data vis-à-vis international policies and regulations, and prevailing standards. The findings regarding data collection and management were particularly illustrative of the challenges inherent in finding a balance between gathering user data for commercial purposes and upholding data privacy safeguards.^55^ While some interviewees saw privacy as the primary concern, pointing to their own policies of not selling data and implementing ‘very strict privacy controls’, others had no qualms admitting that ‘companies need to own the data in order to monetize [it] … and not being able to [do so] would hinder [a] company’s ability to exist’.^56^

The interviews were also quite revealing when it came to how the industry views the user’s consent and the ‘exceptionalism’ of brain data. While some of the interviewees acknowledged that ‘people are a little more personal about brain data’, there was substantial pushback against the idea that brain data could reveal something that would otherwise be indeterminable through existing methods, like tracking online behavior.^57^ Instead, as one interviewee acknowledged, ‘most companies … don’t have data privacy policies and don’t put them in place purposefully to try and not bring [the issues surrounding data privacy] to light because there is no winning that debate’.^58^

### I.C. Public Perceptions of Brain Data: Gauging Sensitivity and Navigating the Ethical Landscape

Ultimately, the utilization of personal data holds significance for the individuals who supply this information. Moon emphasizes the importance of consumer perceptions in shaping health data privacy and usage policies by combining insights from studies investigating consumer preferences for health data sharing.^59^ Mirroring the principles of trust and ethical practice in healthcare delivery, Moon describes consumers as the ‘key stakeholders’ in the policy framework governing health informatics.^60^ Human-centric data governance and decision-making start with individuals’ experiences and perceptions of the data they share.^61^

Notably absent in these discussions is concrete empirical data about how the public perceives the sensitivity of their own brain data. Does the public see their brain data as something uniquely sensitive that can reveal their ‘most intimate identity’, as Donald Kennedy describes?^62^ Do they recognize the unique risks of using consumer neurotechnology? Do they understand the inferences that can be drawn from their brain data? Or, based on their current understanding of neurotechnology, do they regard their brain data as merely another data point, like their online browsing history or birthday?

Here, we present the findings of a nationwide survey conducted in the USA, examining the public’s perception of brain data in comparison to other personal information. While the survey was conducted several years ago, there has not yet been a mass market consumer neurotechnology product launched, although there are several major product launches believed to be on the near-term horizon.^63^ These results inform discussions of law and policy regarding the governance of the data collected by neurotechnology and help to establish a baseline before major product launches in this field. This research offers an empirical grounding of public perceptions and attitudes with respect to the sensitivity of brain data, and their perceptions of unique risks and benefits associated with consumer neurotechnology to further guide ethical, policy, and regulatory discussions on the same.

## II. STUDY DESIGN

Prior studies have explored public perceptions of neural and health data, including data sharing and privacy^64^, neuroimaging data^65^, and data collected using BCI.^66^ The Pew Research Center, a nonpartisan think tank that conducts public opinion polling and analysis, shared a similar but broader objective. In 2014, they conducted a national study that explored Americans’ privacy behaviors and attitudes toward different types of personal information.^67^ Participants were asked to rate the sensitivity of 16 different personal information items, as either ‘not at all sensitive’, ‘not too sensitive’, ‘somewhat sensitive’, or ‘very sensitive’. The study revealed, unsurprisingly, that Americans found certain types of data to be more sensitive than others. Specifically, social security numbers were widely regarded as the most sensitive piece of information, while media tastes and purchasing habits were among the least sensitive pieces of information.^68^ Martin and Nissenbaum explored individuals’ perspectives on data collection and the possible invasion of privacy in their 2017 study.^69^ They cited the Pew Research Center’s survey for its high standing in public discourse and its consistent rating of information sensitivity along a scale.^70^

In addition to adopting the Pew study’s methodology of gaging the public’s perspectives on various types of data, the goal of our study was to identify where brain data lies among the varying degrees of data sensitivity. This study builds upon prior research to understand how information that can currently or could one day be decoded from the data collected by consumer neurotechnology—thoughts in one’s mind, concentration levels, and one’s emotions and feelings—fits into public perceptions of privacy.

### II.A. Methods

We designed a survey modeled on a 2014 Pew research study and included 17 additional information items, detailed in Table 1. We retained all 16 data categories from the Pew study to provide a foundation for understanding public perceptions of data sensitivity and contrast this with the newly introduced items. Given that the Pew study had only one category related to personal health, our questions explored more diverse facets of neural and health data. Items varied from credit score history to alcohol consumption. We defined data as ‘neural’ or ‘brain’ if it directly stemmed from brain function or neurological activity.

**Table 1:** Information items evaluated for sensitivity ratings by survey respondents

Original 16 information items (Pew)	Additional 17 information items
Social security number State of health and medication one takes Content of your phone conversations Content of your email messages Details of your physical location over time Content of your text messages Numbers you have called or texted Your birth date Your relationship history Websites you have visited Searches you have made using search engines Your religious and spiritual views Your friends and what they are like Your political views and the candidate you support The media you like Your basic purchasing habits	Credit score and credit history Intelligence quotient Content of social media accounts Your sexual orientation Your genetic information Your sleep patterns Graphical readout of your brainwave activity Details about your brain health Details about your drug or alcohol use over time The mental images in your mind The mental thoughts in your mind Real-time and accurate details about your mental drowsiness throughout the day Real-time and accurate details about your mental alertness throughout the day Real-time and accurate details about your mental concentration throughout the day Real-time and accurate details about your ability to focus on tasks throughout the day Real-time and accurate details about the mental anxiety you experience throughout the day Real-time and accurate details about the true emotional feelings you experience throughout the day

A brief explanation of the study’s purpose, to collect opinions on privacy in the digital age, was given as contextual information to participants prior to the start of the survey. The survey began with general questions on participants’ feelings about privacy in the digital age. This was followed by three privacy pragmatist measurement questions by Alan Westin, whose contributions have had a lasting effect on the concept of privacy in research and policy. Despite criticisms of his framework, Westin’s three-tiered privacy categories (fundamentalists, pragmatists, and unconcerned) are frequently employed in privacy research, including the Pew and Martin and Nissenbaum studies.^71^ Subsequent sections transitioned to questions on data sensitivity, where participants gaged the sensitivity of various data types using a four-point scale: (i) ‘very sensitive’, (ii) ‘somewhat sensitive’, (iii) ‘not too sensitive’, or (iv) ‘not at all sensitive’. Six demographic questions were included to ensure diverse representation. Question order was randomized in each section to minimize order biases.

#### II.A.1. Recruitment and Sampling

Survey Sampling International, LLC (SSI), a data collection service that specializes in conducting surveys using nationally representative sampling, facilitated recruitment. Their approach, much like GfK Group’s KnowledgePanel from the 2014 Pew study, leveraged online panels, advertisements, social media, and direct emails. While SSI mainly focuses on online recruitment, their methods align with other bioethics studies using internet-only participants.^72^^,^^73^^,^^74^

#### II.A.2. Pilot Testing

Before the full survey, pilot tests were undertaken via Amazon’s Mechanical Turk (MTurk).^75^ These pilots assessed clarity, word choice, and survey duration, laying the groundwork for the main survey’s structure and guiding sample size determination. Rather than a conventional power analysis, our sample size determination was driven by the data from the pilot.

#### II.A.3. Data Collection

Between Dec. 1 and 7, 2017, 2576 participants were surveyed via SSI. The survey, accessible across various digital devices, was hosted on Qualtrics. SSI remained unaware of the specific data. Each survey took about 10 min, and all responses were mandatory. Out of the total, 1126 were discarded due to various reasons, with 1450 responses being analyzed. Demographic details of participants are in Table 2.

**Table 2:** Demographic information of the survey respondents and corresponding US averages

Participant characteristics	Values (%)	2017 US averages (%)^1^^,^^2^^,^^3^^,^^4^
**Gender**		
Male	54.0	48.9
Female	45.7	50.9
Other	0.3	0.2
**Age categories (in months)**		
18–24	13.8	9.7
25–34	16.5	13.7
35–44	17.8	12.7
45–54	19.1	13.4
55–64	15.5	12.7
65 or older	17.3	14.9
**Race categories**		
White	53.0	75.7
Black	13.3	13.9
Hispanic/Latino	10.3	17.6
Asian	5.6	5.4
American Indian/Alaska Native	6.0	0.8
Native Hawaiian/other Pacific Islander	1.9	0.2
Other	9.9	5.4
**Household income categories**		
$15,000 or less	14.8	11.6
$15,000–$24,999	13.1	9.8
$25,000–$34,999	13.9	9.5
$35,000–$49,999	14.2	13.0
$50,000–$74,999	18.3	17.7
$75,000–$99,999	11.9	12.3
$100,000–$149,999	9.2	14.1
$150,000–$199,999	2.8	5.8
$200,000 or more	1.9	6.3
**Marital status**		
Never married	37.3	32.0
Married	45.2	50.4
Separated	1.9	2.0
Divorced	12.7	9.8
Widowed	2.9	5.8
**Political affiliation**		
Democrat	38.2	33.0
Independent	30.8	37.0
Republican	22.1	26.0
Other	8.9	4.0
**Educational level**		
Less than high school	2.8	15.4
High school graduate	19.1	27.5
Some college	34.8	27.2
College graduate	31.7	19.0
Graduate/Professional school	11.6	10.8

^1^Bureau, U.C. (n.d.). *Age and Sex Composition in the United States: 2017*. [online] Census.gov. Available at: https://www.census.gov/data/tables/2017/demo/age-and-sex/2017-age-sex-composition.html.

^2^Bureau, U.C. (n.d.). *2017 Census of Governments – Organization*. [online] The United States Census Bureau. Available at: https://www.census.gov/data/tables/2017/econ/gus/2017-governments.html.

^3^Bureau, U.C. (n.d.). *2013–2017 ACS 5-year Estimates*. [online] Census.gov. Available at: https://www.census.gov/programs-surveys/acs/technical-documentation/table-and-geography-changes/2017/5-year.html.

^4^Pew Research Center (2018). *Trends in party affiliation among demographic groups*. [online] Pew Research Center - U.S. Politics & Policy. Available at: https://www.pewresearch.org/politics/2018/03/20/1-trends-in-party-affiliation-among-demographic-groups/.

Compensation was set at $0.50 per participant, regardless of response inclusion. This compensation model was adopted from MTurk and approved by our Institutional Review Board. We acknowledge ethical considerations concerning payment levels, especially given MTurk’s historical payment trends. Funding for this study was provided by Duke University’s Bass Connections program and the Duke Science, Law, and Policy Lab (SLAP Lab).

#### II.A.4. Data Analysis

All reported analyses were conducted in R (4.3.1; R Core Team, 2023)^76^ with the associated scripts being publicly available.^77^ To discern the underlying variables impacting perceived data sensitivity, an exploratory factor analysis with a ‘promax’ rotation was conducted, using the ‘psych’ package (Revelle, 2020)^78^ in R. Horn’s parallel analysis, run with the ‘paran’ package, helped determine the number of factors in the exploratory factor analysis (Dinno, 2018)^79^ in R. For each individual, we calculated factor scores for three factors: mental/affective, public, and private information. The factor score represents an individual’s perceived sensitivity for one of the three broad categories of information rather than a particular information item. To obtain a factor score for each factor, we only considered information items whose factor loadings were greater than 0.4 (or smaller than −0.4 for negative loadings); factor scores were then computed as the weighted average of those information items. This approach ensured that the factor scores were in the same range as the original ratings (1–4) and allowed for comparisons across factors. To compare the perceived sensitivity of these three information categories (ie factor scores), as well as to investigate its rela tionship to socioeconomic status, linear mixed-effects models (LMMs) were fitted using the ‘lme4’ package (Bates et al., 2015)^80^ in R and significance for fixed effects was assessed using Satterthwaite approximations to degrees of freedom. In all LMMs, continuous variables were standardized, categorical predictors were coded using deviation coding, and participants were included as random intercepts. The ‘emmeans’ package (Lenth, 2020)^81^ was used to probe significant interactions between predictors. All 95% confidence intervals around estimates were computed using parametric bootstrapping with 1000 simulations.

## III. RESULTS

### III.A. Sensitivity Rankings Mirrored the Pew Study

**Figure 1:**
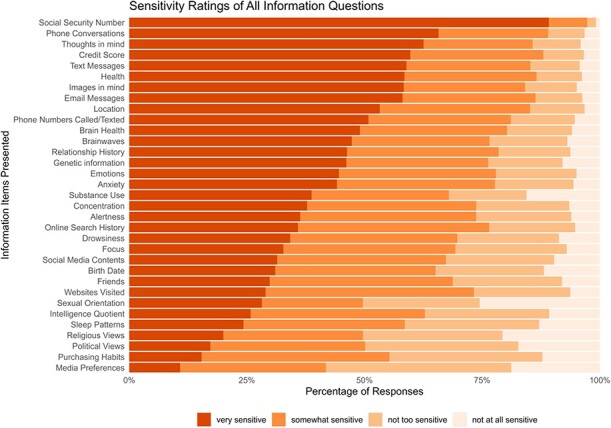
Sensitivity of all information questions.

The top three items from the 16 featured in the Pew survey, rated as ‘very sensitive’, aligned with our study’s findings. Specifically, social security number was identified as the most sensitive, with 89.2% of participants indicating so. Meanwhile, political views, media preferences, and basic purchasing habits were seen as the least sensitive in both studies. Figure 1 illustrates the sensitivity rankings for all 33 information items, while Table 3 presents the mean and standard deviation for these items.

**Table 3:** Mean and standard deviation of sensitivity ratings

Description	Mean	SD
Social security number	3.86	0.46
Phone conversations	3.52	0.77
Credit score	3.44	0.79
Thoughts in mind	3.44	0.83
Health	3.41	0.81
Email messages	3.41	0.81
Text messages	3.4	0.84
Images in mind	3.38	0.86
Location	3.35	0.81
Phone numbers called/texted	3.27	0.89
Brain health	3.23	0.9
Relationship history	3.18	0.91
Emotions	3.18	0.89
Brainwaves	3.17	0.94
Anxiety	3.16	0.9
Genetic information	3.14	0.96
Online search history	3.07	0.86
Concentration	3.05	0.91
Alertness	3.04	0.9
Websites visited	2.96	0.86
Drowsiness	2.95	0.95
Focus	2.95	0.92
Friends	2.91	0.92
Substance use	2.91	1.08
Social media contents	2.89	0.96
Birth date	2.84	1
Intelligence quotient	2.78	0.95
Sleep patterns	2.7	0.98
Purchasing habits	2.58	0.89
Sexual orientation	2.52	1.15
Political views	2.5	0.97
Religious views	2.49	1.03
Media preferences	2.34	0.9

### III.B. Brain Data Items Varied in Their Perceived Sensitivity

Among the 17 new information items, thoughts in one’s mind (62.6%; mean = 3.44, SD = 0.83), one’s credit score (59.7%; mean = 3.44, SD = 0.79), and the content of the images in one’s mind (58.3%; mean = 3.38, SD = 0.86) ranked as the top three ‘very sensitive’ items. While other brain-related data did not make the top five in sensitivity, the content of images in one’s mind did reach the top ten. Items like brain health, brainwave activity, and emotional responses were perceived as moderately sensitive by fewer than half the participants. Other aspects like sleep patterns were deemed ‘very sensitive’ by only 24.3%. Around a third of respondents found concentration (37.8%), alertness (36.3%), drowsiness (34.2%), and focus (32.8%) to be ‘very sensitive’.

### III.C. Overall Brain Data Was Considered Less Sensitive Than Other ‘Private’ Information, but More Sensitive Than ‘Public’ Information

**Figure 2:**
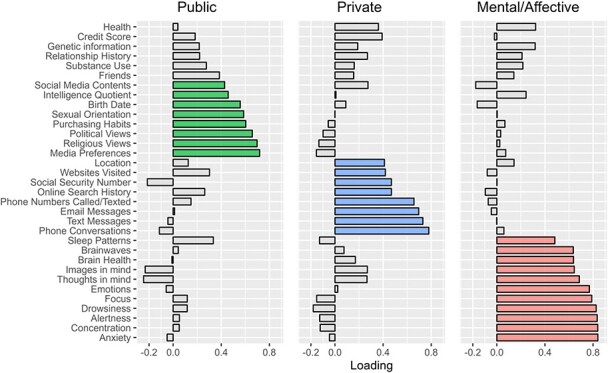
Factor loading for each item.

We conducted an exploratory factor analysis of the participants’ perceived sensitivity of all 33 different information items to determine which information items asked about in the survey had statistically similar patterns of perceived sensitivity. Based on this analysis, we grouped items into three categories. Figure 2 shows the factor loadings for each item. Using a threshold of 0.4, we identified and subsequently analyzed 27 items, excluding the remaining six (friends, substance use, genetic information, relationship history, health, and credit score).

*‘Public*’ category contained information typically shared with others, like social media contents, or shared under certain circumstances, like political or religious views. ‘*Mental/Affective*’ category includes current brain data that can plausibly be collected (brainwaves, sleep patterns, brain health, emotions, focus, drowsiness, alertness, concentration, anxiety) and brain data that could be potentially be collected in the future (eg thoughts in the mind and images in the mind). Lastly, the ‘*Private*’ comprises information that is typically kept confidential, such as one’s online search history, the contents of text messages and phone calls, and one’s social security number. Table 4 itemizes the data in each category.

**Table 4:** Categorization of information items into mental/affective, public, and private categories

Mental/affective information	Public information	Private information
Drowsiness Alertness Focus Concentration Brainwaves Brain health Emotions Anxiety Mental images Mental thoughts Sleep patterns	Political views Media preferences Birth date Religious views Purchasing habits IQ Sexual orientation Social media contents	Text messages Online search history Email messages Physical location over time Social security number Phone conversations Websites visited Phone numbers called/texted

**Figure 3:**
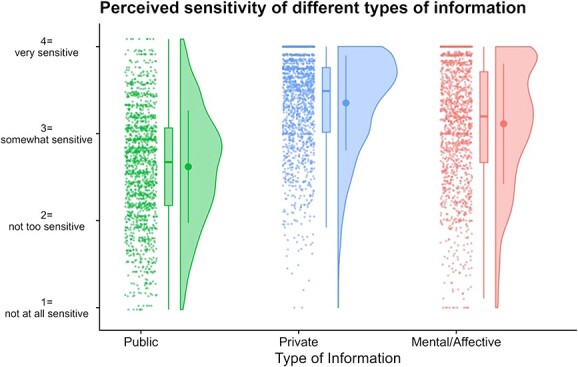
Perceived sensitivity of different types of information.

We used an LMM to analyze the factor scores from these categories. As illustrated in Figure 3, ‘mental/affective’ data (*M* = 3.11, *SD* = 0.69) were perceived as more sensitive than ‘public’ data (*M* = 2.62, *SD* = 0.65; *b* = −0.49, *SE* = 0.02, *df* = 2898, *t* = −30.2, *p* < 0.0001, 95% CI = [−0.52, −0.46]), but less so than ‘private’ information (*M* = 3.35, *SD* = 0.55; *b* = 0.24, *SE* = 0.02, *df* = 2898, *t* = 14.7, *p* < 0.0001, 95% CI = [0.21, 0.27]).

### III.D. Demographic Trends and Sensitivity

We also investigated how demographics influence the perceptions of mental/affective data. Using another LMM, we assessed the influence of participants’ age, gender, education, and income. Age range and income level were treated as continuous variables using the response scales (1–6 and 1–9, respectively; mean-centered and scaled before model fitting), while education and gender were treated as binary variables (‘college degree’ vs. ‘no college degree’, and ‘female’ vs. ‘male’. We excluded a small ‘other’ gender group from our analysis due to its size.

**Figure 4:**
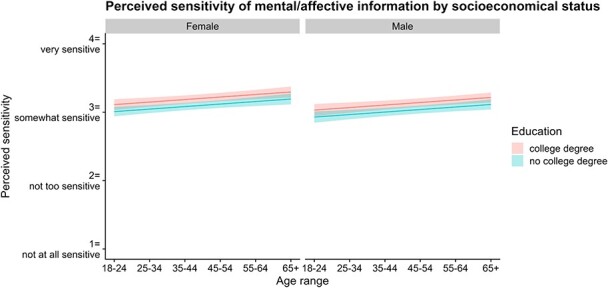
Perceived sensitivity of mental/affective information by socioeconomic status.

Females found mental/affective information to be slightly more sensitive than their male counterparts (*b* = 0.08, *SE* = 0.03, *df* = 2827, *t* = 2.33, *p* = 0.0199, 95% CI = [0.01, 0.15]), while college-educated individuals from both genders tended to view the information as more sensitive than non-college educated respondents (*b* = 0.10, *SE* = 0.04, *df* = 2827, *t* = 2.84, *p* = 0.0046, 95% CI = [0.03, 0.17]). Regardless of gender or education status, as age increased, so too did the perceived sensitivity of mental/affective information (*b* = 0.06, *SE* = 0.02, *df* = 2828, *t* = 3.49, *p* = 0.0005, 95% CI = [0.03, 0.10]). Finally, we did not find perceived sensitivity to vary according to participants’ income level (*b* = 0.03, *SE* = 0.02, *df* = 2827, *t* = 1.54, *p* = 0.1232, 95% CI = [−0.01, 0.06]). Figure 4 compares the factor scores/perceived sensitivity of mental/affective information against these demographics.

### III.E. Tech Adoption and Sensitivity

**Figure 5:**
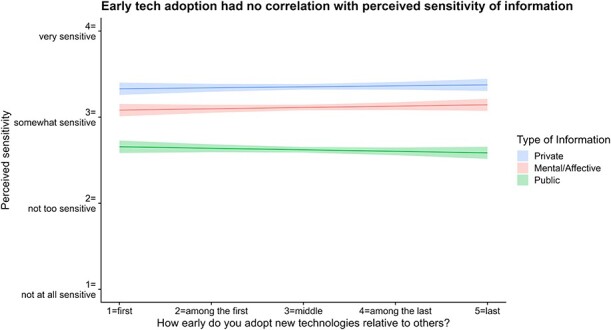
Early tech adoption has no correlation with perceived sensitivity of information.

Lastly, we wanted to see if an individual’s willingness to adopt technology early correlated with their perceived sensitivity. We fitted an LMM to assess whether an individual’s tech adoption status (how early an individual adopts new technology on a 5-point scale) is associated with their perceptions of the sensitivity of the information. Type III ANOVA revealed no significant correlation between how early one adopts new technology and how sensitive one perceives any kind of information (*F* = 0.05, *df* = (1, 1448), *p* = 0.8217) or with the specific type of information (*F* = 2.55, *df* = (2, 2896), *p* = 0.0786; see Figure 5).

## IV. DISCUSSION

In 2017, the same year this study was conducted, an article described the human brain as carrying terabytes of valuable biological and clinical data.^82^ This conceptualization underscored the gravity of harnessing brain data in the digital age, especially as neurotechnology advancements burgeon.

Subsequent literature surrounding neurotechnologies and data privacy has amplified the clarion call for addressing disparities in data governance and protection measures.^83^ For instance, to define brain data, scholars propose: ‘Human brain data are quantitative data about human brain structure, activity, and function’. ^84^ This vast realm ranges from neurobiological metrics, like EEG and fMRI, to rich sociopsychological contexts, especially when merged with non-neural data such as smartphone usage patterns.^85^

This study sought to better understand how the public perceives the sensitivity of brain data. We compared opinions on specific brain data variables (‘mental/affective’ column in Table 4) against other non-neural variables of information (‘private’ and ‘public’ columns in Table 4) to better guide the ethical and legal conversations in this domain.

Contrasting with findings from Schmitt et al., where the US public apparently made little distinction across diverse data categories,^86^ our insights align more with the 2014 Pew study. For example, while ‘thoughts in one’s mind’ was regarded with heightened sensitivity, other facets of brain data like ‘concentration’, ‘focus’, ‘alertness’, and ‘drowsiness’ were nestled lower in the hierarchy, even trailing behind items like ‘relationship history’.

### IV.A. Making Sense of Public Perceptions

We observed an intriguing order of preference among the three data categories; ‘mental/affective’ data, which included our brain data targets, was perceived as more sensitive than ‘public’ information but ranked below ‘private’ data. Significantly, both our findings and the 2014 Pew study ascertained that an individual’s social security number outpaces all forms of mental/affective data in terms of perceived sensitivity.

We believe that there are several potential explanations for the dissonance between public and expert perspectives on the sensitivity of brain data. It is possible that consumers are less familiar with the potential uses of data collected from consumer neurotech devices and, therefore, are less wary of the risks associated with that information being inadvertently exposed. Our survey distribution took place before highly publicized data breaches like the 2018 Cambridge Analytica scandal, which had lasting impacts on the public perceptions of data collection and security. A qualitative study conducted during the aftermath of the Cambridge Analytica scandal asked participants about their understanding of online privacy and personal data.^87^ Not only did their results show that there was a general lack of understanding of how personal data is utilized, but more surprisingly, individuals considered themselves immune to the consequences of breaches affecting their personal data.^88^ One can assume that as neurotechnology becomes more prevalent in society (as exemplified by Elon Musk’s mainstream innovation Neuralink), consumer awareness will also rise with it. However, there remains an evident gap in consumers’ understanding of their brain data. More deliberate efforts in educating the public will enable consumers to better discern the implications of data that neurotechnology can and will capture.

Societal conditioning undeniably shapes our perceptions of ‘sensitive’ information. As observed in the Pew study and ours, social security numbers are universally viewed as highly sensitive,^89^ likely due to the longstanding fears and repercussions associated with identity theft.^90^ By contrast, the ramifications of misused brain data, although potentially calamitous, might not be as ingrained in the public psyche. Neupane et al.’s prediction about tech behemoths like Facebook or Neuralink pioneering thought-to-type technology further underscores the impending challenges to our conventional privacy norms.^91^

Our findings also hint that certain kinds of brain data might intrinsically be viewed as less sensitive than others. For example, generalized affective states like attention and anxiety may be perceived as less intimate than content-rich information like personal thoughts or mental imagery. Such distinctions are pivotal to future studies. If brain data indeed spans a sensitivity spectrum, similar to other personal data types, policies could be crafted to address this spectrum to ensure the balance between promoting innovation, while safeguarding the cognitive liberty of individuals.

Lastly, perceptions of brain data’s utility, especially for altruistic purposes such as advancing brain research, might temper public apprehensions at least with respect to some actors. Schmitt et al.’s insights reveal a preference among the US public for data deployment for ‘the common good’ such as public health research at non-profit organizations, rather than profit-driven purposes such as private organizations or economies.^92^ These findings, albeit not entirely aligned with current US data protection laws, spotlight the need for recalibrating legal safeguards.^93^ Future studies could dive more deeply into these motivations to better inform policy that balances data types and their intended applications.

## V. CONCLUSION

Neurotechnology is rapidly evolving, prompting a need to reconsider our understanding and handling of brain data. Our study has explored public perceptions of different types of brain data and found that sensitivity varies depending on the inferences drawn from the data. However, this study underscores a notable gap: the nature and implications of the raw data itself.

While our analysis largely revolved around inferred data—insights generated from raw brain signals—it hints at a spectrum of data sensitivity, from ‘mental/affective’ data to more direct cognitive processes, suggesting a nuanced approach to data protection. However, raw brain data, which captures basic electrical patterns (like in EMG and EEG), presents distinct challenges.^94^ As technology progresses, such raw data holds potential for diverse personal insights, perhaps beyond the current public understanding.

Several frameworks, including those advanced by Farahany,^95^ the Neurotechnology Ethics Taskforce (NET),^96^ and Ienca et al.,^97^ guide the treatment of brain data, addressing its storage, use, and dissemination. Grounded in public perceptions, these guidelines can be fine-tuned to be more effective. The urgency of brain data protection is gaining global traction. Chile’s recent legal amendments concerning brain data,^98^ and the Organization for Economic Cooperation and Development’s (OECD) 2019 recommendations underscore this momentum.^99^ UNESCO’s recent report^100^ and initiatives^101^ further spotlight the pressing nature of this issue. The focus, however, needs to squarely address the inferences that can be drawn from brain data as well as the nature of the data itself.

If consumer neurotechnology trends toward edge computing and storage, our findings highlight the importance of policies targeting permissible inferences from brain data. Conversely, if edge computing is not the future, there exists a potential disconnect between public perceptions of brain data and the protections required for individual mental privacy.

Our findings emphasize the crucial need to delve deeper into public perceptions of raw brain data and its latent implications. A nuanced approach to regulation is essential, distinguishing between raw and inferred data, with each carrying its own set of immediate and potential implications.

In conclusion, there is an evident need to understand public perceptions surrounding raw brain data and its potential implications. Regulatory strategies should differentiate between raw and inferred data, understanding the unique characteristics of each. Addressing this subject requires an integrated perspective, combining insights from neuroscientists, ethicists, policymakers, data analysts, the public, and a broad and diverse set of stakeholders. While our study provides important insights on public perceptions about inferences that can be drawn from brain data, it also underscores the need for expanded research, especially concerning the less-explored territory of public perception and understanding of the nature of raw brain data.

## Footnotes

1 Kellmeyer, P., *Big Brain Data: On the Responsible Use of Brain Data from Clinical and Consumer-Directed Neurotechnological Devices*. 14 Neuroethics, 83–98 (2021), https://link.springer.com/content/pdf/10.1007/s12152-018-9371-x.pdf.

2 Raghupathi, W., Raghupathi, V. *Big data analytics in healthcare: promise and potential.*  2 Health Inf Sci Syst, 3 (2014). https://doi.org/10.1186/2047-2501-2-3.

3 Farahany, Nita, *The Battle for Your Brain: Defending the Right to Think Freely in the Age of Neurotechnology (St. Martin’s Press 2023); Kreitmair, K.V. Dimensions of Ethical Direct-to-Consumer Neurotechnologies*. 10 AJOB Neuroscience, 152. https://doi.org/10.1080/21507740.2019.1665120.

4 Bedows, A. *Consumer Neurotechnology Volume 1.*  https://loupfunds.com/consumer-neurotechnology-volume-i/ (2018).

5 Farahany, Nita, *The Battle for Your Brain: Defending the Right to Think Freely in the Age of Neurotechnology (St. Martin’s Press 2023); Mittner, M., et al., When the Brain Takes a Break: A Model-Based Analysis of Mind Wandering.*  34 J Neurosci, 16286–16295 (2014), https://doi.org/10.1523/JNEUROSCI.2062-14.2014.

6 Asif, A., Majid, M., & Anwar, S.M., *Human stress classification using EEG signals in response to music tracks.*  107 Comput Biol Med, 182–196 (2019), https://doi.org/10.1016/j.compbiomed.2019.02.015.

7 Scherer, R., et al., *Brain-computer interfacing: more than the sum of its parts.*  17 Soft Computing, 317–331 (2013), https://doi.org/10.1007/s00500-012-0895-4.

8 Mandal, A. and Saxena, N., 2022, May. *SoK: Your mind tells a lot about you: On the privacy leakage via brainwave devices.*  In Proceedings of the 15th ACM Conference on Security and Privacy in Wireless and Mobile Networks (pp. 175–187).

9 Hu, X., et al., *EEG Corelates of Ten Positive Emotions.*  11 Front Hum Neurosci, 26 (2017), https://doi.org/10.3389/fnhum.2017.00026.

10 Mandal, *supra* note 8 at 175.

11 Ienca, M., Haselager, P. & *Emanuel, E. Brain leaks and consumer neurotechnology.*  36 Nat Biotechnol, 805–810 (2018), https://doi.org/10.1038/nbt.4240.

12 Mandal, *supra* note 8*.*

13 Okuda K., *Nissan Brain-to-Vehicle technology redefines future of driving,*  https://usa.nissannews.com/en-US/releases/nissan-brain-to-vehicle-technology-redefines-future-of-driving (2018).

14 Liao, L., et al, *Gaming control using a wearable and wireless EEG-based brain-computer interface device with novel dry foam-based sensors.*  9 NeuroEng Rehabil, 5 (2012), https://doi.org/10.1186/1743-0003-9-5.

15 Meng, J., et al., *Noninvasive Electroencephalogram Based Control of a Robotic Arm for Reach and Grasp Tasks.*  6 Sci Rep, 38,565 (2016), https://doi.org/10.1038/srep38565.

16 Farahany, *supra* note 3.

17 A. Neupane, K. Satvat, M. Hosseini and N. Saxena, *‘Brain Hemorrhage: When Brainwaves Leak Sensitive Medical Conditions and Personal Information,’* 2019, 17th International Conference on Privacy, Security and Trust (PST), Fredericton, NB, Canada, 2019, pp. 1–10, https://doi.org/10.1109/PST47121.2019.8949062.

18
*Id.*

19 Mandal, *supra* note 8 at 178.

20 Kellmeyer, P., *Big Brain Data: On the Responsible Use of Brain Data from Clinical and Consumer-Directed Neurotechnological Devices.*  14 Neuroethics, 83–98 (2021), https://link.springer.com/content/pdf/10.1007/s12152-018-9371-x.pdf.

21 White, T., Blok, E. and Calhoun, V.D., 2022. *Data sharing and privacy issues in neuroimaging research: Opportunities, obstacles, challenges, and monsters under the bed.*  43 Hum Brain Mapp, pp. 278–291.

22 Farahany, *supra* note 2, at 73–75; Kellmeyer, *supra* note 22 at 88.

23 Kellmeyer, *supra* note 22 at 87.

24 Ienca, *supra* note 11 at 808.

25 Kellmeyer, *supra* note 22.

26 Ienca, *supra* note 11 at 810.

27 Farahany, *supra* note 3 at 10.

28 Drew, L., *The ethics of brain computer interfaces.*  571 Nature, S19–S21 (2019), https://doi.org/10.1038/d41586-019-02214-2.

29 Hamilton, J. O’C. *If They Could Read Your Mind.*  Stanford Magazine (Jan/Feb 2004), https://stanfordmag.org/contents/if-they-could-read-your-mind.

30 Mittner, *supra* note 5.

31 Brown, T., Johnson, R., & Milavetz, G., *Identifying Period of Drowsy Driving Using EEG.*  57 Ann Adv Automot Med, 99–108 (2013), https://www.ncbi.nlm.nih.gov/pmc/articles/PMC3861841/.

32 Hu, *supra* note 9.

33 Soares de Aguiar Neto, F., & Garcia Rosa J.L., *Depression biomarkers using non-invasive EEG: a review.* 105 Neurosci Biobehav Rev, 83–93 (2019) https://doi.org/10.1016/j.neubiorev.2019.07.021.

34 Clarke, A.R., Barry, R.J., & Johnstone, S., *Resting state EEG power research in Attention-Deficit/Hyperactivity Disorder: A review update,*  131 Clin Neurophysiol, 1463–1479 (2020), https://doi.org/10.1016/j.clinph.2020.03.029.

35 Yuste, R., et al. *Four ethical priorities for neurotechnologies and AI.*  551 Nature, 159–163 (2017). https://doi.org/10.1038/551159a.

36 Farahany, *supra* note 3, at 25–27.

37 Ienca, *supra* note 11, at 809. *But, see, eg,* Wexler, A. *Separating neuroethics from neurohype.*  37 Nat Biotechnol, 988–990 (2019). https://doi.org/10.1038/s41587-019-0230-z (declaring brain data to be ‘meaningless’ without access to important ‘contextual information, such as the content and timing of a stimulus presented to a subject,’ in comparison to ‘the data that we generate on a daily basis’ such as ‘e-mail messages, browser queries, passwords, financial information, call data and GPS information.’).

38 Goering, S., et al., *Recommendations for Responsible Development and Application of Neurotechnologies.*  Neuroethics (2021), https://doi.org/10.1007/s12152-021-09468-6.

39 Kellmeyer, P., *Big Brain Data: On the Responsible Use of Brain Data from Clinical and Consumer-Directed Neurotechnological Devices.*  14 Neuroethics, 83–98 (2021), https://link.springer.com/content/pdf/10.1007/s12152-018-9371-x.pdf.

40 Farahany, *supra* note 3 at 29.

41
*Id*.

42
*Id*.

43 Ienca, *supra* note 11, at 807.

44 Ienca, *supra* note 11, at 809.

45 Hallinan, D., Schütz, P., Friedewald, M. and De Hert, P., 2014. *Neurodata and neuroprivacy: Data protection outdated?*  12 Surveill Soc, pp. 55–72.

46 McCall, I.C. and Wexler, A., 2020. *Peering into the mind? The ethics of consumer neuromonitoring devices. In Developments in Neuroethics and Bioethics* (Vol. 3, pp. 1–22). Academic Press.

47 Gerber, N., Reinheimer, B. and Volkamer, M., 2019. *Investigating People’s Privacy Risk Perception.*  Proc. Priv Enhancing Technol, 2019, pp. 267–288.

48
*Id.*

49
*Id.*

50 Kreitmair, *supra* note 3, at 153.

51 Jwa, A.S. and Poldrack, R.A., 2022. *Addressing privacy risk in neuroscience data: from data protection to harm prevention.*  9 J Law Biosci, p. lsac025.

52 Wexler, A. and Reiner, P.B., 2019. *Oversight of direct-to-consumer neurotechnologies.*  Science, 363 (6424), pp. 234–235. Vancouver.

53
*Id.*

54 Minielly, N., Hrincu V. & Illes, J., *Privacy Challenges to the Democratization of Brain Data.*  23 iScience, 101134 (2020), https://doi.org/10.1016/j.isci.2020.101134.

55
*Id.* at 2.

56
*Id.*

57
*Id.*

58
*Id.*

59 Moon, L.A., 2017. *Factors influencing health data sharing preferences of consumers: A critical review.*  6 Health Policy Technol, pp. 169–187.

60
*Id.* at 183.

61 Kennedy, H., Oman, S., Taylor, M., Bates, J. and Steedman, R., 2021. *Public understanding and perceptions of data practices: A review of existing research*.

62 Hamilton, *supra* note 30.

63 Farahany, *supra* note 3, Chapter 1.

64 Ford, E., Curlewis, K., Wongkoblap, A. and Curcin, V., 2019. *Public opinions on using social media content to identify users with depression and target mental health care advertising: Mixed methods survey.*  6 JMIR Mental Health, p. e12942.

65 Chandler, J.A., Sun, J.A. and Racine, E., 2017. *Online public reactions to fMRI communication with patients with disorders of consciousness: Quality of life, end-of-life decision making, and concerns with misdiagnosis.*  8 AJOB Empirical Bioethics, pp. 40–51.

66 Sample, M., Sattler, S., Rodriguez-Arias, D., Blain-Moraes, S. and Racine, E., *Do Publics Share Experts’ Worries About Brain-Computer Interfaces? A Trinational Survey on the Ethics of Neural Technology*.

67 Madden, M., *Public Perceptions of Privacy and Security in the Post-Snowden Era*, http://www.pewinternet.org/2014/11/12/public-privacy-perceptions/ (2014).

68
*Id.* at 31.

69 Martin, K. & Nissenbaum, H., *Measuring Privacy: An Empirical Test Using Context to Expose Confounding Variables.*  18 Colum Sci & Tech L Rev, 176–218 (2017).

70
*Id.*

71 Urban, Jennifer M. and Chris Jay Hoofnagle. *The Privacy Pragmatic as Privacy Vulnerable* (July 9, 2014), Symposium on Usable Privacy and Security (SOUPS 2014) Workshop on Privacy Personas and Segmentation (PPS), July 9–11, 2014, Menlo Park, CA, UC Berkeley Public Law Research Paper No. 2514381.

72 Smith, A. and Brenner, J., 2012. *Twitter use 2012. Pew internet & American life project*, 4, pp. 1–12.

73 Purcell, K., 2011. *Search and email still top the list of most popular online activities* (pp. 9–8). Washington, DC: Pew Internet & American Life Project.

74 Schmit, C., Giannouchos, T., Ramezani, M., Zheng, Q., Morrisey, M.A. and Kum, H.C., 2021. *US privacy laws go against public preferences and impede public health and research: survey study.*  23 J Med Internet Res, p. e25266.

75 Benbunan-Fich, R., 2023. *To pay or not to pay? Handling crowdsourced participants who drop out from a research study.*  25 Ethics Inf Technol, p. 34.

76 R Core Team R: A Language and Environment for Statistical Computing, Version 4.3.1, [https://www.R-project.org/] (https://www.R-project.org/).

77 Huang, S. (2023). USPerceptionBrain. [online] GitHub. Available at: https://github.com/Huang-Shenyang/USPerceptionBrain [Accessed Sep. 7, 2023].

78 Revelle, W. *psych: Procedures for Psychological, Psychometric, and Personality Research*, Version 2.3.12, [https://CRAN.R-project.org/package=psych] (https://CRAN.R-project.org/package=psych).

79 Dinno, A. *paran: Horn's Test of Principal Components/Factors*, Version 1.5.2, [https://CRAN.R-project.org/package=paran] (https://CRAN.R-project.org/package=paran).

80 Bates, D., Mächler, M., Bolker, B. and Walker, S. *Fitting Linear Mixed-Effects Models Using lme4*, 67 Journal of Statistical Software, 1–48 (2005).

81 Lenth, R. *emmeans: Estimated Marginal Means, aka Least-Squares Means*, Version 1.5.0, [https://CRAN.R-project.org/package=emmeans] (https://CRAN.Rproject.org/package=emmeans).

82 Landhuis, E., 2017. *Neuroscience: Big brain, big data.*  Nature, 541(7638), pp. 559–561.

83 Farahany, *supra* note 3; Ienca, M., Fins, J.J., Jox, R.J., Jotterand, F., Voeneky, S., Andorno, R., Ball, T., Castelluccia, C., Chavarriaga, R., Chneiweiss, H. and Ferretti, A., 2022. *Towards a governance framework for brain data.*  15 Neuroethics, p. 20.

84
*Id.*

85
*Id.*

86 Schmit, *supra* note 77.

87 Hinds, J., Williams, E.J. and Joinson, A.N., 2020. *‘It wouldn’t happen to me’: Privacy concerns and perspectives following the Cambridge Analytica scandal.*  Int J Hum Comput Stud, 143, p. 102498.

88
*Id.*

89 Madden, *supra* note 67.

90 Irshad, S. and Soomro, T.R., 2018. *Identity theft and social media.*  18 Int J Comp Sci Netw Secur, pp. 43–55.

91
*Id.*

92 Schmit, *supra* note 77, at 10.

93 Schmit, *supra* note 77, at 1–2.

94 Farahany, *supra* note 3, at 23–25.

95 Farahany, *supra* note 3.

96 The NET was formerly known as the Morningside Group and ‘comprises neuroscientists, neurotechnologists, clinicians, ethicists and machine learning engineers.’ Yuste, *supra* note 22, at 160.

97 Ienca, *supra* note 11.

98 Montes, R., *Chile, the NeuroRights International Lab.* (2020), https://nri.ntc.columbia.edu/news/chile-neurorights-international-lab-originally-spanish.

99 Organization for Economic Co-Operation and Development, *Recommendation of the Council on Responsible innovation in Neurotechnology,* 0457, 8 (2019), https://legalinstruments.oecd.org/en/instruments/OECD-LEGAL-0457.

100 UNESCO, *Unveiling the Neurotechnology Landscape: Scientific Advancements Innovations and Major Trends* (2023), available at https://unesdoc.unesco.org/ark:/48223/pf0000386137.

101
https://www.unesco.org/en/articles/ethics-neurotechnology-unesco-leaders-and-top-experts-call-solid-governance.

