# Erratum to: U.S. public perceptions of the sensitivity of brain data

**DOI:** 10.1093/jlb/lsae009

**Published:** 2024-06-01

This is an erratum to: Shenyang Huang, Umika Paul, Shikhar Gupta, Karan Desai, Melinda Guo, Jennifer Jung, Beatrice Capestany, William D Krenzer, Dylan Stonecipher, Nita Farahany, U.S. public perceptions of the sensitivity of brain data, *Journal of Law and the Biosciences*, Volume 11, Issue 1, January-June 2024, lsad032, https://doi.org/10.1093/jlb/lsad032.

In the originally published article the fourth author was incorrectly listed as Karen Desai. This has been corrected to read Karan Desai.

